# A Study of the Current Scenario of the Obstetrics and Gynecology Residency during the COVID-19 Pandemic

**DOI:** 10.1055/s-0043-1772181

**Published:** 2023-08-18

**Authors:** Marina Martinelli Sonnenfeld, Alexandre Massao Nozaki, Anelise Silva de Genova, José Ricardo Weitz Ristow, Tamiris Ferreira de Moura, Rayanne Pereira Cabral, João Victor Almeida Garcia, Camila Abdias Guimarães, André Luiz Malavasi Longo de Oliveira, Rossana Pulcineli Vieira Francisco

**Affiliations:** 1 Centro Universitário Faculdade de Medicina do ABC, Santo André, SP, Brazil; 2 Centro de Referência da Saúde da Mulher, Hospital Pérola Byington, São Paulo, SP, Brazil; 3 Associação Lar São Francisco de Assis na Providência de Deus, Hospital Regional de Presidente Prudente, Presidente Prudente, SP, Brazil; 4 Pontifícia Universidade Católica de Campinas, Campinas, SP, Brazil; 5 Universidade Estadual Paulista “Júlio de Mesquita Filho,” Botucatu, SP, Brazil; 6 Hospital Maternidade Leonor Mendes de Barros, São Paulo, SP, Brazil; 7 Hospital Municipal Doutor José de Carvalho Florence, São José dos Campos, SP, Brazil; 8 Faculdade de Medicina, Universidade de São Paulo, São Paulo, SP, Brazil

**Keywords:** internship and residency, gynecology, COVID-19, medical education, residência médica, ginecologia, COVID-19, educação médica

## Abstract

**Objective** To analyze the impact of the COVID-19 pandemic on the residency of gynecology and obstetrics in São Paulo.

**Methods** Cross-sectional study developed by representatives of residents of the Association of Gynecology and Obstetrics of the State of São Paulo (SOGESP, in the Portuguese acronym). Data were collected from questionnaires applied to gynecology and obstetrics residents registered on the SOGESP website in February 2022. The interviewees answered about the repercussions of the pandemic on medical residency and whether they had technical and psychological support during the period.

**Results** A total of 247 questionnaires were collected from residents of gynecology and obstetrics. The residents had an age of 28.3 ± 3 years old, and most of them were female (88.4%). The displacement to COVID care was reported by 62.34% of the students, but only 35.6% reported completely adequate personal protective equipment and only 7.7% reported complete theoretical and technical instruction to support these patients. Almost all of the interviewees stated that the gynecology sector was the most affected. The majority of the interviewees considered that the second-year residents had the greatest loss, and more than half of the residents in the 1 ^st^ and 2 ^nd^ year said they wished to give up their residency during the pandemic. More than 80% of the residents reported online theoretical classes and/or presentation of articles, reinforcing the fact that virtual activities gained a greater space within the medical residency.

**Conclusion** The pandemic impacted the residency in greater proportion in outpatient clinics and gynecological surgeries, also interfering with the physician's desire to continue with the program.

## Resumo

**Objetivo** Analisar o impacto pandemia de COVID-19 sobre a residência de ginecologia e obstetrícia do Estado de São Paulo.

**Métodos** Estudo transversal desenvolvido por representantes dos residentes da Associação de Ginecologia e Obstetrícia do Estado de São Paulo (SOGESP). Foram coletados dados de questionários aplicados aos residentes de ginecologia e obstetrícia cadastrados no site da SOGESP em fevereiro de 2022. Os entrevistados responderam sobre repercussões da pandemia sobre a residência médica e se tiveram suporte técnico e psicológico durante o período.

**Resultados** Foram levantados 247 questionários de residentes de ginecologia e obstetrícia. Os residentes apresentaram idade de 28,3 ± 3 anos, sendo a maioria do sexo feminino (88,4%). O deslocamento para “covidários” foi referido por 62,34% dos avaliados, porém somente 35,6% referiram equipamento de proteção individual completamente adequado e apenas 7,7% referiram instrução completa teórica e técnica para o suporte destes pacientes. Quase a totalidade dos entrevistados afirmou que o setor de ginecologia foi o mais afetado. A maioria dos entrevistados considerou que o os residentes do segundo ano foram os que tiveram maior prejuízo, sendo que mais da metade dos residentes do 1° e 2° ano afirmou ter desejado desistir da residência durante a pandemia. Mais de 80% dos residentes referiram aulas teóricas e/ou apresentação de artigos online, reforçando o fato de que as atividades virtuais ganharam um espaço maior dentro da residência médica.

**Conclusão** A pandemia impactou nas residências em maior proporção nos ambulatórios e cirurgias ginecológicas, interferindo também sobre o desejo do médico de seguir com o programa.

## Introduction

The worldwide pandemic of COVID-19, which surprised the world in the year 2020, was a landmark in practically all professional branches. Several companies had to close their doors or move their activities to “home-office,” while a minority continued to work face-to-face, as in the case of healthcare professionals. As for medical residency, the reality varied depending on the specialty, due to the cancellation of elective surgeries and outpatient clinics considered non-essential. In addition, many resident doctors were allocated to areas of exclusive care of patients with COVID-19. In the case of gynecology and obstetrics, it was no different. Although more than a year has passed since the beginning of the pandemic, it is still not possible to assess the impact of the pandemic on residents from an academic, physical, and psychological point of view.

The medical residency has proven to be the most effective method for medical training, with Brazil currently being the third country with the highest percentage of specialist doctors in gynecology and obstetrics. In 2019, of the 53,776 resident physicians in training, 4,609 were in gynecology and obstetrics. Of these, 1,243 were in their 1 ^st^ year and were directly impacted by COVID-19 in the following two years of residency. It is important to emphasize that 33.9% of all resident physicians are concentrated in the state of São Paulo, which represents approximately one-third of the national total. Within the expected workload, according to the National Commission of Medical Residency (CNRM, in the Portuguese acronym), residents of gynecology and obstetrics should have 48 hours in practical activities and 12 hours in theoretical activities weekly, and the residency should include the clinical and surgical areas, with competencies that contribute to the effective management of situations, problems, and dilemmas in the specialty, besides developing a critical-reflexive thought in regard to the medical literature, making residents progressively more accountable and independent. It is not known, however, whether or not physicians were trained in all the recommended skills during the pandemic. 1 2

In a study with 148 residents from 18 countries, published by the American Confederation of Urology in 2020, for example, 82% responded that activities within the urology department were significantly reduced and 15% responded that activities were completely canceled. In addition, 75% responded that their surgical training was completely affected by the pandemic. On the other hand, a North American study that assessed the impact of the pandemic on emergency medical education identified the impacts on clinical training, teaching education, and concentration and emotional mental states. In the case of gynecology and obstetrics, the activities include theoretical and practical content within various subspecialties, with modules of care considered essential, such as the obstetrics and oncology sector, as well as services considered non-essential, such as climacteric, family planning, and urogynecology outpatient clinics, for example. 3 4

Considering a broad view of the situation, it is still unclear what the impacts of the pandemic are on the training of gynecologists and obstetricians. There are still no large-scale studies within this specialty in the country. The Obstetrics and Gynecology Association of the state of São Paulo has been supporting the residencies through monthly virtual educational content in nine regional offices, in addition to changing the format of congress from the traditional face-to-face to an electronic format. Considering the residents in training, the purpose is to map out the repercussions of the pandemic period on this public, including physical, structural, academic, and psychic aspects, as well as to establish a profile of this population.

The main objective of the present study was to analyze the impact of the COVID-19 pandemic period in the academic, physical, and psychological domains of first, second, and third-year obstetrics and gynecology residents in the state of São Paulo. The second objective was to profile the residents evaluated, as well as the percentage of residents who were affected by COVID-19.

## Methods

A cross-sectional analytical study was performed by researchers representing gynecology and obstetrics residents belonging to the Obstetrics and Gynecology Association of the State of São Paulo (SOGESP, in the Portuguese acronym). All data were collected from questionnaires administered to first, second, and third-year residents of the Gynecology and Obstetrics residency program in the state of São Paulo who answered a virtual questionnaire during the stipulated period in February 2022.

Its high social relevance is emphasized for addressing the impacts of the pandemic on the training of professionals who will enter the medical market.

The data of the participants was kept secret and confidential. At no time will the name be disclosed in any phase of the research, which guarantees anonymity, and the results will be disclosed in such a way as not to identify the individuals.

Participants from all years of the medical residency program in obstetrics and gynecology, whose institutions belong to the state of São Paulo, and whose residency program coordination authorized the administration of the questionnaires were included.

Participants who had already graduated or who had not initiated specialization were excluded, as well as participants of medical residency in specialties other than gynecology and obstetrics, or who are not attending residency registered in the state of São Paulo. Additionally, residents of institutions whose coordination did not authorize the implementation of the questionnaire were excluded, thus totaling 45 institutions of residency in obstetrics and gynecology out of the 63 listed in the state of São Paulo.

In February 2022, questionnaires were administered to gynecology and obstetrics residents regarding the impact of the COVID-19 pandemic on residency programs and the clinical data of the residents.

The study was performed virtually, through the Website and the Associação de Ginecologia e Obstetrícia do Estado de São Paulo ( http://sogesp.com.br ). The resident registered at SOGESP received an invitation by e-mail beforehand to answer the survey.

The questionnaire comprised a total of 24 questions, in which data were recorded, such as whether the resident had COVID during the pandemic, classifying it as mild, moderate, or severe. The physician was asked whether the resident was assigned to care for patients with COVID-19 in adult emergency rooms (labeled as “Covidarium”) or intensive care units (ICUs). The participant classified the impact of the pandemic on medical residency as “None,” “Minimal,” “Partial” or “Total” considering outpatient care, gynecological surgeries, obstetric procedures, and lectures. Residents were asked which year had the greatest losses as a result of the pandemic in residency (first, second, or third year), and if he/she is in favor of an additional year for residents most affected by the pandemic. From an academic point of view, the resident was also asked if there was a proposal to make up for the lost activities in the residency due to the pandemic. Once these considerations were made, the participant was asked about their intention to subspecialize and if this was influenced by the period experienced.

Considering the psychological domain, the participant was asked if they received psychological support for issues other than the pandemic and if they considered quitting the residency during the pandemic. In addition, the participant was asked about their vacation time from the medical residency program, whether it was affected in any way by the COVID-19 pandemic, and whether there was any proposal for replacement as well as for the case of elective internships.

From the standpoint of COVID-19 patient care, residents were asked whether the medical residency supplied adequate personal protective equipment (PPE) to care for contaminated patients and whether theoretical and technical preparation was provided, which was classified as “None,” “Minimal,” “Partial,” or “Total”. In line with the rationale of protection from exposure to the virus, we asked about the provision of vaccination against COVID-19 by the institution.

Following the investigation of academic repercussions during the pandemic period, the participant answered whether they have a preference between face-to-face and virtual congress. In addition, the resident was asked about their participation in the development of any scientific article during the pandemic and whether they consider that the pandemic interfered with their scientific production.

Finally, the resident answered if they consider that their specialization was more affected in the obstetrics or gynecology sector, ending with an open question for suggestions to make up for the damage caused by the pandemic in medical residency.

The present study included the questionnaires answered by the resident physicians who met the selection criteria (convenience sample). The characteristics of the participants were presented descriptively (minimum values, maximum values, numbers, percentages, median, and standard deviation [SD]).

Data were distributed using the Kolmogorov-Smirnov test. Chi-squared, Fisher, or Student *t* -tests were used depending on the nature of the variables. For non-normal distribution data, nonparametric tests were used. P values < 0.05 were considered statistically significant.

The data obtained were organized in electronic spreadsheets of Microsoft Excel 2018 version 1910 software (Microsoft Corporation, Redmond, WA, USA).

The present study was approved by the research ethics committee of the *Centro de Estudos e Pesquisas Dr. João Amorim* - CEJAM (CAAE 50512821.9.0000.9107).

## Results

**Fig. 1:**
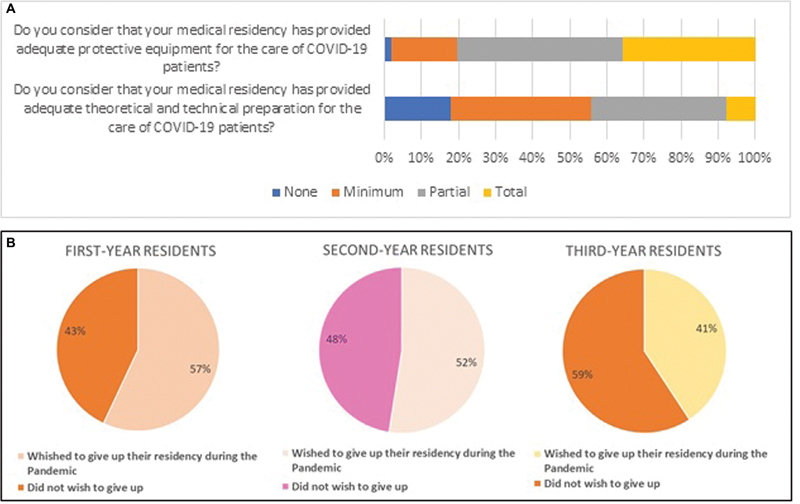
( **A** ) Analysis of residents' preparation for care of patients with COVID-19. ( **B** ) Analysis of residents according to desire to quit residency program during the pandemic.

Considering the inclusion criteria, 248 questionnaires from gynecology and obstetrics residents were included and 1 questionnaire was excluded as it belonged to a medical residency that was not from the state of São Paulo, resulting in 247 remaining questionnaires, which would correspond to 19.4% of the ∼ 1,274 residents of the specialty in the state of São Paulo during the year 2021. The physicians were distributed according to the regional offices of SOGESP in the state. The majority of respondents belonged to medical residency programs in the city of São Paulo (Headquarters) (46.5%), compared with the regions of ABC Paulista (15.9%), Campinas (13.8%), Santos (8.1%), Ribeirão Preto (7.7%), Midwest (4.0%), Vale do Paraíba (1.6%), São José do Rio Preto (1.6%) and Presidente Prudente (0.8%). The residents were 28.3 years old and most of them were female (88.4%). Regarding residency year, 86 people were in their 1 ^st^ year (34.8%), 80 people were in their 2 ^nd^ year (32.4%), and 81 people were in their 3 ^rd^ year of residency (32.8%). Among the physicians evaluated, 153 participants (61.9%) reported having had COVID during the pandemic. Among the physicians who had a confirmed diagnosis, 53.4% classified the disease as “mild” and 8.5% of those evaluated classified it as “moderate.” It is important to emphasize that none of the residents approached reported having had a severe form of COVID, and ∼ 90% of the interviewees reported that the vaccine was available during their residency. Assignments within the residency to care for patients affected by COVID-19 were confirmed by 62.34% of those evaluated. In addition, 42.9% reported direction to care for patients in the ICU affected by COVID-19. Considering the technical preparation for this type of care with the supply of PPE, the residents were questioned according to Fig. 1 .

The residents were evaluated regarding their intention to give up their medical residency during the pandemic according to the year of their residency, as shown in Fig. 1 . It is noteworthy that among the physicians who reported that they had wished to give up their residency during the pandemic, 73.6% reported that their residency had no psychological support service.

**Fig. 2:**
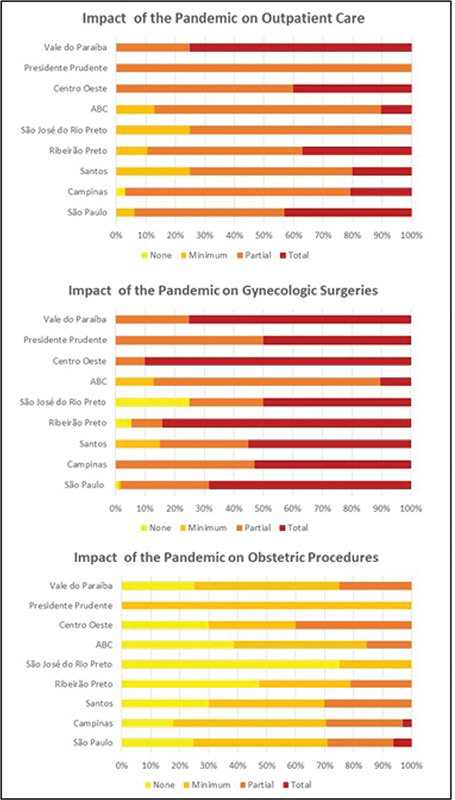
Analysis of the impact of the pandemic on outpatient care, gynecological surgeries and obstetric procedures.

Within the scope of the impact of the pandemic on medical residency, the questionnaires were stratified according to the regions of SOGESP in the state of São Paulo, according to Fig. 2 . When asked, 96.3% of the interviewees stated that residency was more affected in the gynecology sector compared with obstetrics.

**Fig. 3:**
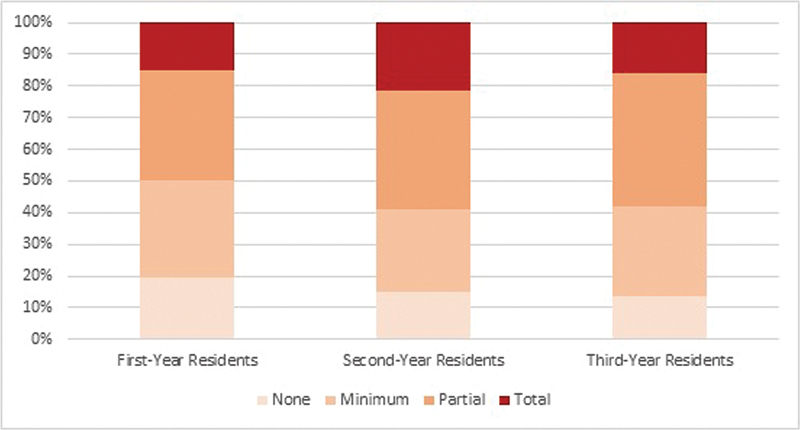
Analysis of the impact of the pandemic on theoretical classes.

It is emphasized that only 31% of the residents reported that there was a proposal for making up for the lost activities. Still, 94.7% of those surveyed expressed the desire to subspecialize, and only 18.1% of those interviewed said that the pandemic interfered with their choice. When asked, 79.3% expressed the desire to work in obstetrics after the conclusion of medical residency, 94.7% expressed the desire to maintain contact with gynecology and 64.0% expressed the desire to work with both areas. Within the lost theoretical-practical content, almost 80% of the residents who have the possibility of doing the optional external internship reported that there was a loss regarding this benefit. Considering the activities performed remotely, in video classes on the computer, in 82.5% of the residencies there was a reference to theoretical classes and/or presentation of articles online. Although virtual classes were a convenient format, 60.3% of the physicians said they preferred the face-to-face format. However, this number increased to 80.5% when they were asked about their preference regarding congresses ( Fig. 3 ).

## Discussion

First of all, when analyzing data, it is noteworthy that the great majority of interviewees belonged to the city of São Paulo. This is consistent with the fact that most medical residences in the state of São Paulo are currently concentrated in the capital of the state or neighboring cities (27%), not to mention large cities such as ABC Paulista (which ranked second in the questionnaires, or Campinas [which ranked third]).

Regarding the gender identity of the evaluated residents, women predominated in the present study with 88.4%. These data are compatible with the number found in other gynecology and obstetrics medical residences around the world; for example, with an English study from 2022, finding 85% of female residents, while another Italian study from 2020 found 81%. What is the reason for this majority of female doctors within gynecology? There are still no articles in the literature assessing this fact and the studies show no preference on the part of patients regarding the gender of the attending physician. It is worth mentioning here that Brazil is undergoing a process of feminization of medicine and that among the physicians who currently practice the profession, the majority are still male, but among those who are ≤ 29 years old, the majority are now female. The age found among the interviewees was consistent with other studies, emphasizing that while in Brazil the medical residency in gynecology and obstetrics lasts 3 years, in other countries it can take up to 5 years. 5 6 7 8 9

The pandemic required great resilience from the medical staff, directly affecting the education of obstetrics and gynecology residents, who had to balance the clinical care of patients while learning specialty skills. Among the significant findings of the present study, > 60% of those evaluated reported having had the disease, and none of the residents reported a severe form of COVID. This can be accounted for the fact that the medical population was one of the first to receive the vaccination (emphasizing that almost all of the interviewees reported receiving the vaccine through their residency). Moreover, another point attributable to the good response to the disease could be related to the young age of this population, 28.3 years old, which is known to relate to better outcomes within COVID infection, considering the lower rate of associated comorbidities for this group. 10

Among the main factors responsible for the impact of the pandemic on medical residency, we can highlight the cancellation of elective surgeries and the reassignment of residents to clinical departments, the so-called “covidariums,” which may have compromised both theoretical and surgical learning. In a study of general surgery residents in Greece, for example, it was found that 54.8% of physicians who were reassigned to clinical areas reported that their surgical skills were negatively affected by the pandemic, a figure that fell to 24.7% of residents who were not reassigned. 11

In the present study, it was identified that 62.34% of those evaluated were directed to this type of care, while only 35.6% of the interviewees reported having received completely adequate PPE, and only 7.7% reported complete theoretical and technical instruction to care for these patients. This result was below the results of a study performed with gynecology and obstetrics residents in Italy, in which 56.1% reported having received adequate safety equipment, and 79.6% reported having been well informed about prevention and management protocols for these patients. 6

Regarding the impact of the pandemic on the psychological condition of residents, the correlation between the vulnerability to psychological pathologies and the frequency of contact with patients affected by COVID-19 is well known, resulting in physicians even considering dropping out of residency. When we asked this question, we expected a good number of 3 ^rd^ -year residents to have expressed this intention, since they experienced the pandemic in the 2 ^nd^ and 3 ^rd^ years of residency, in 2020 and 2021, which are the two years of most surgical practice, especially in the field of gynecology. However, the majority of the interviewees (44.5%) considered that 2 ^nd^ -year residents were the ones who suffered the greatest loss academically due to COVID-19, behind 1 ^st^ -year residents (39.7%) and 3 ^rd^ -year residents (15.8%). It is emphasized that for 1 ^st^ - and 2 ^nd^ -year residents, more than half of the respondents stated that they wished to quit residency during the pandemic, while in the case of 3 ^rd^ -year residents, the rate was lower. It is worth pointing out here that it is not only the pandemic that affects the resident's intention to quit training, but also the pressure of the work itself. The first year of residency is the most affected at this point. These results reinforce the fact that medical residency programs should ensure healthy communication to promote mental health for physicians by providing psychological support in or out of pandemic situations. 12 13 14

Regarding the impact of the pandemic on teaching practices, as seen in Fig. 2 , it can be said that the conflict was bigger within gynecologic surgery and outpatient clinics compared with obstetric procedures, considering all regions of the state, which was expected to some extent. Considering that we cannot stop obstetric procedures even in adverse conditions, such as in the pandemic, 96.3% of the interviewees affirmed that residency was more affected in the Gynecology sector than in Obstetrics. In all regional offices of SOGESP, ≥ 70% of the residents considered the impact of the pandemic on outpatient clinics to be partial to total, and more alarmingly, in 8 of the 9 regional offices evaluated, half or more of the respondents considered the impact on gynecological surgeries to be total. This interference of the pandemic may have practical repercussions, creating professionals lacking the confidence to perform surgical procedures in their postresidency practice. According to the residents, repercussions on theoretical activities were lower than on practical ones, which was unanimous among the interns during the 3-year internship; however, 50% of the interviewees considered that the impact was from partial to total. This was largely due to the online platforms that emerged in the pandemic, which was reported by 82.5% of the respondents, and in some cases, these activities continued online even after the global situation improved. Despite the pandemic and COVID-19 interfering with teaching practices, only 18% of the respondents reported that this period interfered with their intention to pursue subspecialty training, a number close to the 20% found in a study of London obstetrics and gynecology residents who reported an impact of the pandemic on career choice. The number of residents willing to continue working in obstetrics (79.3%) was below the 84% observed in the Canadian study and lower than the 94.7% found for gynecology. 5 15 16

The weaknesses of the present study are: it is a cross-sectional study, with all the limitations attributed to it; the survey was performed only with residents from the state of São Paulo, with no possibility of extending these results to other states or even countries. The strengths of the study are a large number of interviewees, from different regions of the state, with this being one of the first studies on the impact of the pandemic on obstetrics and gynecology residents in Brazil.

## Conclusion

The pandemic had an impact on gynecology and obstetrics residency in the state of São Paulo. It was greater on practical activities such as outpatient clinics and gynecological surgeries than on obstetrics and theoretical classes. It also affected the mental health of residents.
